# The humidity level matters during the desiccation of Norway spruce somatic embryos

**DOI:** 10.3389/fpls.2022.968982

**Published:** 2022-07-29

**Authors:** Lucie Fischerová, Lenka Gemperlová, Milena Cvikrová, Ildiko Matušíková, Jana Moravčíková, Zuzana Gerši, Jiří Malbeck, Jan Kuderna, Jana Pavlíčková, Václav Motyka, Kateřina Eliášová, Zuzana Vondráková

**Affiliations:** ^1^Laboratory of Biologically Active Compounds, Institute of Experimental Botany of the Czech Academy of Sciences, Prague, Czechia; ^2^Department of Ecochemistry and Radioecology, University of Ss. Cyril and Methodius in Trnava, Trnava, Slovakia; ^3^Department of Biotechnologies, University of Ss. Cyril and Methodius in Trnava, Trnava, Slovakia; ^4^Department of Biology, University of Ss. Cyril and Methodius in Trnava, Trnava, Slovakia; ^5^Laboratory of Mass Spectroscopy, Institute of Experimental Botany of the Czech Academy of Sciences, Prague, Czechia; ^6^Laboratory of Hormonal Regulations in Plants, Institute of Experimental Botany of the Czech Academy of Sciences, Prague, Czechia

**Keywords:** somatic embryogenesis, *Picea abies* (L.) Karst, desiccation, polyamines, β-1, 3-glucanases, chitinases, germination

## Abstract

In Norway spruce, as in many other conifers, the germination capacity of somatic embryos is strongly influenced by the desiccation phase inserted after maturation. The intensity of drying during desiccation eminently affected the formation of emblings (i.e., seedlings developed from somatic embryos). Compared to non-desiccated embryos, the germination capacity of embryos desiccated at 100% relative humidity was about three times higher, but the reduction of relative humidity to 95 and 90% had a negative effect on the subsequent embryo development. The water loss observed in these embryos did not lead to an increase in lipid peroxidation, as shown by malondialdehyde levels. Another metabolic pathway in plants that mediates a response to abiotic stresses is directed toward the biosynthesis of polyamines (PAs). The activities of PA biosynthetic enzymes increased steadily in embryos during desiccation at 100% relative humidity, whereas they decreased at lower humidity. The total content of free PAs in the embryos gradually decreased throughout desiccation. The increase in free putrescine (Put) and perchloric acid-insoluble Put conjugates was observed in embryos desiccated at lower humidity. These changes were accompanied to some extent by the transcription of the genes for the PA biosynthesis enzymes. Desiccation at 100% relative humidity increased the activity of the cell wall-modifying enzymes β-1,3-glucanases and chitinases; the activities of these enzymes were also significantly suppressed at reduced humidity. The same pattern was observed in the transcription of some β-1,3-glucanase and chitinase genes. Desiccation treatments triggered metabolic processes that responded to water availability, suggesting an active response of the embryo to the reduction in humidity. A positive effect was demonstrated only for desiccation at high relative humidity. Some of the physiological characteristics described can be used as markers of inappropriate relative humidity during somatic embryo desiccation.

## Introduction

Plants are commonly exposed to various environmental stresses (drought, high temperature, and cold) during their development. Optimal and efficient stress responses at different stages of plant development are prerequisites for a good plant survival strategy (He et al., 2018). In conifers, various stress treatments have a fundamental impact on seed germination and thus the yield and quality of seedlings. The study of embryo development facilitates the explanation of seed strategy during the pre-germination phase of development (Leprince et al., 2017) with the aim of optimizing cultivation protocols. However, the genetic diversity and seasonal development of temperate tree seeds complicate the research on the effects of stressors on zygotic embryo development. These problems can be overcome using somatic embryogenesis (SE) as an experimental system. It is important to note, that SE lacks megagametophyte and is very much influenced by artificial media and conditions applied. The cultivation conditions are, moreover, very different compared to the natural conditions of zygotic embryo development. Nevertheless, SE provides the possibility of studying the effects of selected factors prior to more complex studies of their combined effects.

Somatic embryogenesis is considered an advantageous technique for breeding programs and *in vitro* plant propagation, including conifers (e.g., Park, 2002; Egertsdotter et al., 2019). Under suitable conditions, a relatively high number of embryos can be obtained during all SE steps (i.e., induction, proliferation, maturation, and germination); however, optimal conditions differ among conifer species and among individual embryogenic lines. Qualities of mature embryos by means of both morphological and biochemical status influence germination frequency and limit embryo conversion to plants (Högberg et al., 2001; Le et al., 2021). The period of desiccation at high relative humidity, which is often interposed between maturation and germination, is stressful, but can still have a positive effect on embryo germination (Lelu et al., 1995; Find, 1997; Hay and Charest, 1999; Hazubska-Przybyl et al., 2015; Jing et al., 2017). Our recent integration analysis of embryos desiccated at high relative humidity showed changes in carbohydrates, phytohormones, and proteome (Eliášová et al., 2022), indicating both physiological maturation and accumulation of stress-related molecules. The beneficial effect of desiccation treatment is related to the intensity of drying, while the optimal humidity during desiccation varies among species and genotypes (Roberts et al., 1990, 1991; Attree et al., 1995; Dronne et al., 1997; Jones and Van Staden, 2001).

Drop in humidity during desiccation affects the dehydration of somatic embryos and thus the rate of osmotic stress. Osmotic stress is an important signal that promotes the transformation of somatic embryos from morphological maturity to physiological maturity (Liao and Juan, 2015) and controls embryo development toward germination and subsequent conversion to seedlings in many plant species (Von Aderkas and Bonga, 2000; Yakovlev et al., 2016), including conifers (Klimaszewska et al., 2016). In a model system of Picea abies SE, several protocols have been developed to achieve physiological maturity prior to germination. This can be achieved by desiccation treatment at high relative humidity (Eliášová et al., 2022) or by low temperatures (Tikkinen et al., 2018b).

In a model system of *Picea abies* SE several protocols for achieving pre-germination physiological maturity were developed, it can be achieved both by application of desiccation at high relative humidity treatment (Eliášová et al., 2022) or by low temperatures (Tikkinen et al., 2018b).

Dehydration induced by desiccation is associated with the production of reactive oxygen species (ROS), which are potentially harmful to all cellular components and negatively affect cellular metabolic processes (Van Breusegem et al., 2001; Mailloux and Harper, 2011). Consequently, antioxidant defense in embryos is activated by a complex of enzymatic and non-enzymatic systems that include low-molecular-mass antioxidants (e.g., ascorbate, glutathione), ROS scavenging enzymes (e.g., catalase, peroxidase, etc.), and polyamines (PAs). Among the osmotically active substances generated during dehydration, PAs play an important role (Vondrakova et al., 2010; Vondráková et al., 2015; Cvikrova et al., 2016; Salo et al., 2016; Eliášová et al., 2018; Chen et al., 2019). PAs are ubiquitous biogenic amines that are involved in various cellular functions in all organisms (Handa et al., 2018; Igarashi and Kashiwagi, 2019). It has been suggested that PAs may mitigate the damage caused by abiotic stress (especially drought) by contributing to osmotic adjustment, maintenance of membrane stability, scavenging of free radicals, and regulation of stress-responsive genes (Groppa and Benavides, 2008; Kusano et al., 2008; Velarde-Buendia et al., 2012; Alcázar et al., 2020). Biosynthesis of the three most abundant PAs – putrescine (Put), spermidine (Spd), and spermine (Spm) – is initiated in plants either by direct decarboxylation of ornithine by the enzyme ornithine decarboxylase (ODC; EC 4.1.1.17), or by decarboxylation of arginine by the enzyme arginine decarboxylase (ADC; EC 4.1.1.19) *via* agmatine and N-carbomoylputrescine intermediates. Another essential enzyme in PA synthesis is S-adenosylmethionine decarboxylase (SAMDC; EC 4.1.1.50), which is required for the formation of the aminopropyl group in Spd and Spm (for a review, see Michael, 2016). In plant cells, PAs occur as free molecules, covalently bound to small molecules, especially hydroxycinnamic acids (conjugated PAs), or bound to high molecular mass substances such as hemicelluloses and lignin, and in small amounts to proteins (bound PAs; Igarashi and Kashiwagi, 2015; Mustafavi et al., 2018).

In addition to their important role in responding to stresses, PAs play a crucial role in embryo development, and their content is specific to developmental stages (Monteiro et al., 2002; Minocha et al., 2004; Silveira et al., 2004). Similar to PAs, some cell wall modifying enzymes such as β-1,3-glucanases (Dong and Dunstan, 1997; Doxey et al., 2007) and chitinases (Grover, 2012) have shown functional diversification from various cellular to stress responses (or *vice versa*). β-1,3-glucanases (EC 3.2.1.39) catalyze the cleavage of 1,3-β-D-glucoside bonds in β-1,3-glucans, a major structural component of fungal cell walls (Leubner-Metzger and Meins, 1999). In plants, β-1,3-glucans include callose, which is involved in various biological processes related to plant growth, development and stress responses (Chen and Kim, 2009). Chitinases (EC 3.2.1.14) primarily catalyze the hydrolytic cleavage of the β-1,4-glycoside bond of chitin. In addition, arabinogalactan proteins are hydrolyzed by plant chitinases (Van Hengel et al., 2002), and plant cell wall glycoproteins containing N-acetylglucosamine are also considered an endogenous substrate for chitinases (Dyachok et al., 2002). Most plant chitinases and β-1,3-glucanases are expressed as a defense mechanism during pathogen infection and wounding and they are representatives of two major protein families associated with pathogenesis (Sudisha et al., 2012). It is well documented that they are also involved in plant responses to abiotic stresses, e.g., osmotic, salt, cold, wounding, and heavy metal stresses (for review see, e.g., Balasubramanian et al., 2012; Vaghela et al., 2022). Cell wall composition and flexibility are important for cell differentiation during embryogenesis. In black pine SE, the correlation between the presence of certain chitinase or glucanase isoforms and the embryogenic potential of different embryogenic cultures has been described (Fráterová et al., 2013). The chitinase and β-1,3-glucanase genes are developmentally regulated during SE in *Picea glauca*, and chitinases also regulate differentiation of early somatic embryos in *P. abies* (Wiweger et al., 2003). A basic chitinase secreted by embryogenic tissues of *Pinus caribaea* acts on arabinogalactan protein extracted from the same cell line, thereby generating organogenesis factors (Domon et al., 2000). Chitinases are also involved in regulation of growth and development through programmed cell death; chitinase-mediated autolysis played a role in two waves of programmed cell death required during somatic embryo development in *P. abies* (von Arnold et al., 2005). Chitinases have been suggested as potential markers for the process of SE (Gulzar et al., 2020).

It has been described that β-1,3-glucanases play an important role in seed maturation and also later during germination when they initiate the rupture of the seed testa (Leubner-Metzger, 2005). However, they are also involved in the early stages of somatic embryo development. This may be related to the fact that the acquisition of embryogenic competence is influenced by the physical isolation of the cell from other cells, which can be achieved by the absence of plasmodesmatal contact (Bonga et al., 2010). The deposition and degradation of callose in the neck region of plasmodesmata is one of the cellular control mechanisms regulating their permeability (Zavaliev et al., 2011) and has been demonstrated in the embryogenic cells of several plants (reviewed in Namasivayam, 2007). The deposition of callose was more pronounced in lines producing somatic embryos than in blocked lines of Brazilian pine (Navarro et al., 2022).

Desiccation at high relative humidity (approximately 100%) is routinely used in conifer SE (Roberts et al., 1990; Bomal and Tremblay, 1999; Jing et al., 2017; Vondrakova et al., 2018). Recently, the beneficial effect of desiccation on achieving metabolic maturity of Norway spruce embryos has been demonstrated (Eliášová et al., 2022). The optimal levels of osmotic stress vary among conifer species and need to be tuned empirically. Here we exposed Norway spruce somatic embryos to lower humidity during the desiccation phase to reveal how germination and morphology of emblings are affected. We were interested in investigating possible indicators of osmotic stress during desiccation of embryos at reduced humidity, such as membrane peroxidation rate and accumulation of abscisic acid (ABA), but especially metabolism of PAs and activities of cell wall-modifying β-1,3-glucanases and chitinases. We were also interested in uncovering the level at which the presumed stress response is regulated and therefore we monitored the expression of relevant genes.

## Material and methods

### Plant material and stress application

In the first series of experiments, we investigated the importance of the desiccation step for the development of emblings from 12 embryogenic lines of Norway spruce [*P. abies* (L.) Karst]. Embryogenic cultures were induced in our laboratory in August 2020 from immature zygotic embryos. Induction, proliferation, and maturation proceeded under conditions described in Vagner et al. (2005) and Gemperlova et al. (2009). During proliferation the cultures were grown on GD media (Gupta and Durzan, 1986), solidified with 0.75% agar (Sigma–Aldrich, Steinheim, Germany). The pH was adjusted to 5.8 prior to autoclaving. The medium was supplemented with 0.21 mM cefotaxime (Sefotak, Valeant Czech Pharma, Prague, Czechia), 5 μM 2,4-dichlorophenoxyacetic acid (2,4-D), 2 μM kinetin, 2 μM 6- benzylaminopurine and 30 g/l sucrose (Duchefa, Haarlem, Netherlands). All phytohormones and organic components other than sucrose were diluted separately. The solutions were then filter-sterilized and added to cooled autoclaved media. The embryogenic cultures were maintained by weekly subculturing into Magenta vessels (Magenta corporation, Chicago, IL, United States) containing 40 ml of fresh medium. Cultures were kept in darkness at 24 ± 1^°^C.

In order to initiate the maturation, the cytokinins and auxin in media were replaced with 20 μM ABA (Sigma–Aldrich, Steinheim, Germany) and 3.75% polyethylene glycol 4000 (PEG, Sigma–Aldrich, Steinheim, Germany), pH was adjusted to 5.8 before autoclaving. ABA and all of the organic components, except sucrose, were separately prepared and diluted, filter-sterilized and added to the cooled, autoclaved media. The PEG solution was autoclaved separately and added to the medium after autoclaving. During maturation, the cultures were subcultured onto membrane rafts (Osmotek, Rehovot, Israel) in Magenta vessels with fresh liquid maturation medium at subcultivating intervals of 1 week. Cultures were kept in darkness at 24 ± 1^°^C for 5 weeks.

In the first series of experiments, morphologically fully mature somatic embryos were collected after 5 weeks of maturation, embryos were characterized by apical meristem surrounded with elongated cotyledons, hypocotyl and radicle with root apical meristem and the root cap, and with protoderm on the embryo surface, as described in Eliášová et al. (2018). Embryos were either immediately germinated or treated with high relative humidity desiccation. Desiccation treatment was performed according to Vondrakova et al. (2018). During this treatment, embryos were placed on the dry filter paper laid in open small dishes situated in large Petri dish (18 cm in diameter) with several paper layers wetted by sterile water. Large dishes were covered with lids, sealed with parafilm, and kept under a light regime of 12 h photoperiod (70 μM/m^2^/s) at 18 ± 1°C for 3 weeks. Under this setting, the relative humidity inside Petri dishes reaches near 100%. In all experiments, treatment of embryos under 100% humidity served as a control variant.

For germination, both groups of somatic embryos (immediately after maturation or desiccation treatment at high relative humidity) were placed into the Magenta vessels containing 1/4 strength GD (Gupta and Durzan, 1986) phytohormone-free medium solidified with 0.75% (w/v) agar (Sigma–Aldrich, Germany), and supplemented with 1% (w/v) sucrose and activated charcoal (0.4%; w/v). Cultivation was performed under a 12-h photoperiod at 24 ± 1^°^C (120 μM/m^2^/s). The development of the emblings was evaluated after 3 and 6 weeks, respectively. Images of 15–20 emblings of each variant and embryogenic line were acquired using a Nikon SMZ 1500 stereomicroscope and a Nikon DS-5M color camera (Tokyo, Japan). Images were processed using the NIS-Elements AR 3.2 analysis system (Laboratory Imaging, Prague, Czechia). The number of emblings that formed primary roots, emblings with formed terminal bud, and the number of malformed emblings were counted.

In the second series of experiments, the effect of drought stress during desiccation on development of embryos/emblings was tested using selected embryogenic lines of Norway spruce (AFO 541 from AFOCEL – France, and 6 lines induced in our laboratory). Induction, proliferation and maturation proceeded as described above. Morphologically fully mature somatic embryos (after 5 weeks of maturation) were subjected to different desiccation treatments. In the control variant, conditions of the treatment were the same as in the first series of experiments – filter paper layers in large Petri dishes, where embryos were deposited in small dishes, were wetted with sterile water (near 100% relative humidity). Lower humidity in Petri dishes was achieved when a supersaturated solution of Na_2_HPO_4_.12H_2_O (95% relative humidity) or a supersaturated solution of ZnSO_4_ (90% relative humidity) was used for paper wetting instead of water (Roberts et al., 1990). Because lower humidity was lethal to embryos when applied throughout the desiccation period (results not shown), the desiccation was divided into two phases. During the first half of desiccation (11 days), embryos were maintained under lower humidity; subsequently, the small Petri dishes with embryos were transferred to 100% relative humidity for the rest of desiccation (next 11 days). Control embryos were exposed to 100% relative humidity throughout the process (22 days). The conditions for subsequent germination were as described above. After 3 weeks of germination, the development of emblings was evaluated. Images of 15–20 emblings from each variant (treatment and genotype) were acquired using a Nikon SMZ 1500 stereomicroscope equipped with a Nikon DS-5M color camera. The length of roots, hypocotyls, and whole shoots was measured using a NIS-Elements image analysis system AR 3.2.

**FIGURE 1:**
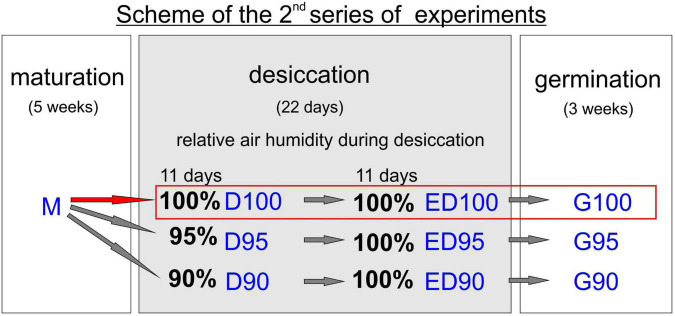
Design of the second series of experiments and sampling. Mature somatic embryos (5 weeks of maturation, M) were subjected to three different systems of desiccation. Control embryos (in red) were exposed to 100% relative humidity throughout the process (sampling D100 and ED100). The second and third groups of embryos were subjected to 95 and 90% relative humidity, respectively, during the first half of desiccation (sampling D95 and D90) and then transferred to 100% relative humidity (sampling ED95 and ED90). The germination rate and morphology of embryos desiccated at different relative humidity levels were examined (G100, G95, and G90). The designation of all samples according to this scheme is used in the following text.

The samples for biochemical analyses were collected at the end of maturation (M), after 11 days of desiccation treatment (D), and at the end of desiccation treatment (ED). All samples were frozen in liquid nitrogen and stored at -80^°^C until analyses. The scheme of the second series of experiments is presented in Figure 1, where the sampling points and sample designations are shown.

### Determination of dry weight of embryos

At each sampling point, samples consisting of approximately 30 embryos were collected and weighed to determine their fresh weight (FW). The samples were collected in triplicate. They were then dried at 80^°^C and continuously weighed to a constant weight to obtain the final dry weight (DW). Dry matter values were expressed as %DW = DW/FW × 100 and concomitantly the water content was calculated as (FW-DW)/DW and is therefore expressed as g H_2_O/g DW.

### Malondialdehyde assay

The malondialdehyde (MDA) content of the samples was determined using the NWLSS-malondialdehyde assay kit (Cat. No. NWK-MDA01, Northwest Life Science Specialties, LLC, Vancouver, Canada) as described in detail by Cvikrova et al. (2012). The assay is based on the reaction of MDA with thiobarbituric acid, which forms an MDAeTBA2 adduct that absorbs light strongly at 532 nm. The amount of MDA in the sample was determined using a five-point standard curve.

### Abscisic acid and abscisic acid metabolite determination

Analysis of the content of ABA and its metabolites in embryos was performed as described previously (Prerostova et al., 2021). Phytohormones were extracted from frozen samples (approximately 10 mg FW aliquot) using 50 μl cold extraction solvent (1M formic acid). After addition of isotope-labeled standards and homogenization, phytohormones were separated on Kinetex EVO C18 column (2.6 μm, 150 × 2.1 mm, Phenomenex, Torrance, CA, United States). Hormone analysis was performed using an LC-MS system consisting of UHPLC 1290 Infinity II (Agilent, Santa Clara, CA, United States) set in multiple reaction-monitoring mode, using the isotope dilution method. Data acquisition and processing were performed using Mass Hunter B.08 software (Agilent). Phytohormone concentrations were calculated as the amount per 1 *g* of DW.

### Ornithine decarboxylase, arginine decarboxylase, and S-adenosylmethionine decarboxylase activity assays

The activities of ODC (EC 4.1.1.17), ADC (EC 4.1.1.19), and SAMDC (EC 4.1.1.50) were determined using the radiochemical method developed by Tassoni et al. (2000) and modified according to Gemperlová et al. (2005). Samples were extracted in three volumes of ice-cold 0.1M Tris–HCl buffer, pH 8.5, containing 2 mM b-mercaptoethanol, 1 mM EDTA and 0.1 mM pyridoxal phosphate, and centrifuged at 20,000 × *g* for 30 min at 4^°^C. Aliquots (0.1 ml) of both supernatant (soluble fraction) and resuspended pellet (particulate fraction) were used to determine ODC and ADC activity. Enzyme activity assays were performed by measuring the ^14^CO_2_ evolution from 7.4 kBq L-[1-^14^C]ornithine (1.92 GBq mmol^–1^, Amersham Pharmacia Biotech, United Kingdom) or 7.4 kBq L-[U-^14^C]arginine (11.5 GBq mmol^–1^, Amersham Pharmacia Biotech), for ODC and ADC, respectively, in the presence of 2 mM unlabeled substrate during a 1.5-h incubation at 37^°^C. ^14^CO_2_ was entrapped in hyamine hydroxide and the radioactivity was counted on liquid scintillation analyzer, Tri-Carb 2900TR, Packard. To determine SAMDC activity samples were homogenized in three volumes of 0.1M phosphate buffer, pH 7.6 containing 2 mM β-mercaptoethanol and 1 mM EDTA, and centrifuged at 20,000 × *g* for 30 min at 4^°^C. The supernatant and resuspended pellet (0.1 ml aliquots) were incubated separately with 3.7 kBq [1-^14^C]S-adenosylmethionine (2.15 GBq mmol^–1^, Amersham Pharmacia Biotech) in the presence of 2.8 mM unlabeled substrate and 3 mM Put. ^14^CO_2_ evolution was measured for 1 h at 37^°^C. The radioactivity was counted on liquid scintillation analyzer, Tri-Carb 2900TR, Packard.

Total protein content of the samples was measured by the Bradford method using bovine serum albumin as a standard (Bradford, 1976).

### Polyamine analysis

Embryos were ground in liquid nitrogen and extracted for 1 h at 4^°^C with 1 ml of 5% (w/v) perchloric acid (PCA) per 100 mg fresh mass of tissue. 1,7-Diaminoheptane was added as an internal standard, and the extracts were centrifuged at 21,000 *g* for 15 min. PCA-soluble free PAs were determined in one half of the supernatant. The remaining supernatant and the pellet were acid hydrolyzed in 6N HCl for 18 h at 110^°^C to obtain PCA-soluble and PCA-insoluble PA fractions. PA standards (Sigma-Aldrich, Czechia) and extracted free, PCA-soluble and insoluble PAs were benzoylated according to the method of Slocum et al. (1989), extracted by distilled diethyl ether and after evaporation stored in -20^°^C until analysis. Detection and quantification of benzoylamines were carried out using an HPLC-MS system consisting of an HTS-Pal auto-sampler with cooled sample stack (CTC Analytics, Zwingen, Switzerland), Rheos 2200 quaternary HPLC pump (Flux Instruments, Basel, Switzerland), Delta Chrom CTC 100 Column oven (Watrex, Praha, CR), and TSQ Quantum Ultra AM triple-quad high-resolution mass spectrometer (Thermo Electron, San Jose, United States) equipped with an electrospray interface. The dried extract was re-dissolved in 5 ml of 80% (v/v) methanol, and 0.5 ml was taken for further analysis and filtered using a 0.2 μm Micro-Spin centrifugal filter (GRACE, Deerfield, IL, United States). An aliquot of 5 μl was injected onto a Kinetex 2.6 μm C_18_ 100 Å HPLC column, 50 × 2.1 mm (Phenomenex, Torrance, United States) and analyzed by elution with a binary gradient (water/acetonitrile) starting at 20% (v/v) and ending at 50% (v/v) acetonitrile. The residual sample was removed from the column by increasing the acetonitrile content to 90% (v/v) for 6 min, and then the column was equilibrated at 20% (v/v) acetonitrile for 6 min before the next injection. The mass spectrometer was operated in the positive SRM (single reaction monitoring) mode and 2–4 transitions were monitored for each compound. The most intensive ion was used for quantification, the others for identity confirmation. PAs were quantified using a multilevel calibration graph with diaminoheptane as the internal standard.

### Zymography of chitinases and β-1,3-glucanases

Total proteins were extracted from embryos according to Hurkman and Tanaka (1986). The enzymes of chitinases and β-1,3-glucanases were detected in polyacrylamide gels as described previously (Galusova et al., 2015). Briefly, protein aliquots (10 μg) were separated in 12.5% (w/v) polyacrylamide mini-gels (Mini-PROTEAN Tetra Cell Apparatus, Bio-Rad) containing enzyme substrate; 0.01% (w/v) glycol chitin was used for chitinases and 0.01% (w/v) laminarin (Sigma) for β-1,3-glucanases. Samples were not boiled before loading. The separation of acidic/neutral and basic/neutral proteins under native conditions was performed according to the method of De Bolle et al. (1991) at 120 V for 3 h at 8^°^C. After electrophoresis, proteins were renatured overnight in 50 mM sodium acetate buffer (pH 5.0), 1% (v/v) Triton X-100. Chitinase fractions were stained with 0.01% (w/v) Fluorescent Brightener 28 (Sigma) in 250 mM Tris–HCl (pH 8.9) for 15 min, and detected by illumination with UV light. Fractions with β-1,3-glucanase activity were visualized as red bands by boiling the gels in 200 ml of 1M NaOH containing 0.3 *g* of 2,3,5-triphenyltetrazolium chloride (Sigma) in a water bath for 5–10 min. After photography (BioDoc-It 210 Imaging System, UVP, California, United States), gel images were processed using Scion Image Software^1^ (Galusova et al., 2015). Background-corrected integrated density (ID) of chitinase bands was calculated in areas of constant size: ID = *N* × (mean-background) where *N* is the number of pixels in the selected area and the background is the modal gray value (pixels). The sizes of individual isoforms were determined based on the co-separated molecular standard.

### Quantitative real-time PCR

Relative transcript levels of the genes of interest were analyzed by real-time PCR. RNA was isolated from 100 mg of frozen embryos using the RNeasy Plant Kit (Qiagen) and treated with DNaseI (Thermo Scientific). cDNA was prepared using Revert Aid First Strand cDNA Synthesis Kit (Thermo Scientific). Quantitative RT-PCR was performed in 12 μl PCR mix containing 6 μl of PCR MasterMix (Generi Biotech), 3.5 μl of nuclease-free water, 0.5 μl of mixture of forward and reverse primers (initial concentration 10 mM) and 2 μl of cDNA. The alpha-tubulin gene from *P. abies* (GenBank: X57980.1) was used as a reference. Primers for the genes of interest were designed based on either gene homology: β-1,3–glucanase (GenBank: L49179.1), putative class I chitinase (GenBank: AY450922.1), class IV Chia4-Pa chitinase (GenBank: AY270016.1), or literature data – ADC, SAMDC, spermidine synthase (SPDS), and SPMS (Vuosku et al., 2012). Primer specificity was demonstrated by PCR using genomic DNA of *P. abies* (DNeasy Plant Mini Kit, Qiagen); PCR products were purified using QIAquick PCR Purification kit (Qiagen) and sequenced (Eurofins Genomics). Sequences were then compared with database data (NCBI). Relative transcript levels were analyzed by the modified 2^–ΔΔ*CT*^ method using individual amplification efficiency for each gene (Livak and Schmittgen, 2001; Schefe et al., 2006) and compared relative to expression levels at the end of maturation (prior to desiccation) as a reference (value1).

### Statistical analysis

Three or four independent experiments were performed, each with at least two biological replicates. Results are presented as means ± standard deviations. The data were analyzed by one-way analysis of variance (ANOVA). The means were compared using the Tukey test, and differences between means with *P*-values less than 0.05 were considered significant. The statistical package SigmaPlot 14.0 was used for all analyses.

## Results

### The effect of high relative humidity treatment on the germination of somatic embryos

**FIGURE 2:**
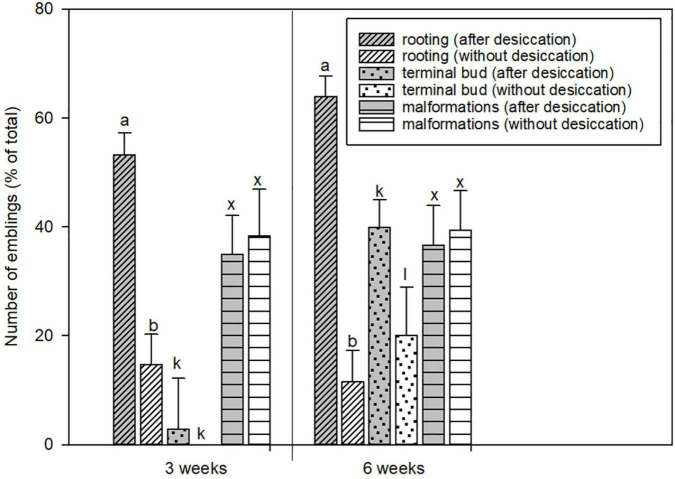
The comparison of the development of somatic embryos from the first series of experiments with or without the desiccation phase. The proportion of the emblings with primary root, with growing terminal bud, and malformed emblings was evaluated after 3 and 6 weeks of germination in response to desiccation (gray columns) and without treatment (white columns). Values are expressed as means ± standard deviations (*n* = 12) from two independent experiments. Different letters above columns indicate a statistically significant difference at *p* ≤ 0.05.

In the first series of experiments, the effect of treatment with 100% relative humidity was tested in 12 embryogenic lines. Germination of a total of 400 emblings derived from treated and untreated embryos was compared based on the percentage of emblings with developed primary root and/or terminal bud and on the number of malformed emblings (Figure 2). The results showed that the high (near 100%) relative humidity treatment promoted embryo rooting and consequently terminal bud formation. Nearly 70% of the treated embryos were able to form primary roots, in contrast to less than 20% of the untreated embryos. The positive effect of the treatment on terminal bud formation was evident after 3 weeks of germination, and was significant after 6 weeks of germination. The treatment did not cause significant damage to the embryos, as the number of malformations was similar in both groups of emblings. Therefore, the conditions of the 3-week 100% relative humidity treatment were used as control conditions in the second series of experiments, where we tested the effect of reduced relative humidity.

### The effect of reduced relative humidity treatments on embling morphology

**FIGURE 3:**
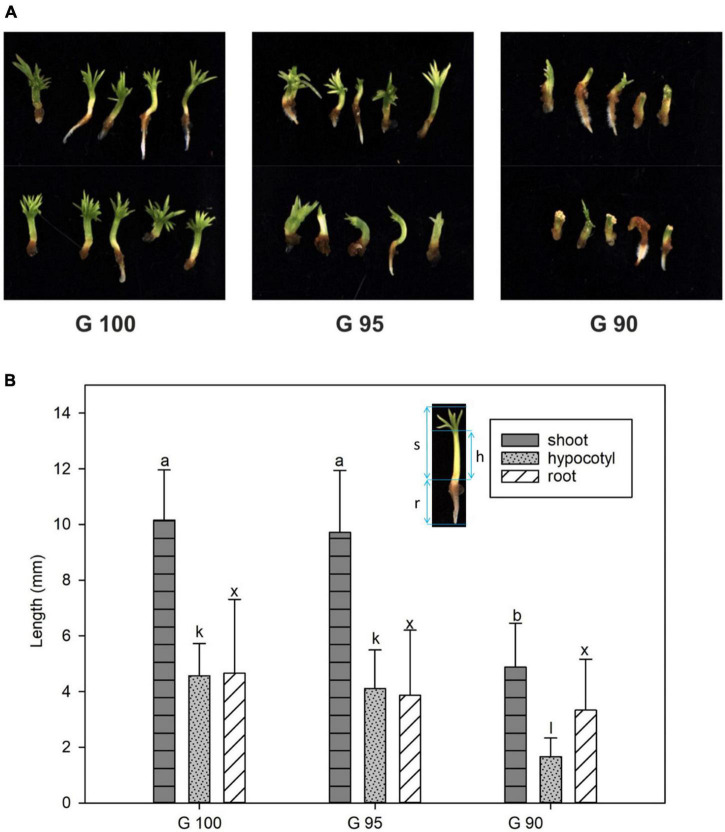
Morphology of 3-week-old emblings developed from embryos desiccated at different relative humidity levels (G100, G95, and G90). **(A)** Overview of emblings; **(B)** Length of whole shoots (dark gray columns), hypocotyls (light gray columns), and primary roots (white columns). The image of the embling inserted in the graph **(B)** shows the measured parts – shoot (s), hypocotyl (h), and primary root (r). Values are given as means ± standard deviations (*n* = 8) of four independent experiments. Different letters indicate a significant difference at *p* ≤ 0.05.

In the second series of experiments, the 3-week-old emblings derived from control embryos (exposed to 100% relative humidity; G100), had green cotyledons, an elongated hypocotyl, and a primary root, just as in the first series of experiments. Water deprivation during the first 11 days of desiccation treatment affected the development of emblings. Hypocotyls often thickened and did not elongate, and cotyledons were poorly developed (Figure 3A). The degree of these malformations depended on stress intensity. While moderate moisture deficit (95% relative humidity) during desiccation had no significant effect on G95 emblings, desiccation at the lowest relative humidity (90%) resulted in significantly inhibited growth of G90 emblings in terms of shoot, hypocotyl and/or root lengths (Figure 3B).

Root formation was less affected than shoot growth; 33% of G95 emblings and 77% of G90 emblings were able to form roots compared to 53% in G100, and root length remained comparable to the G100 control emblings (Figure 3B).

### The changes in water content of embryos during desiccation

**FIGURE 4:**
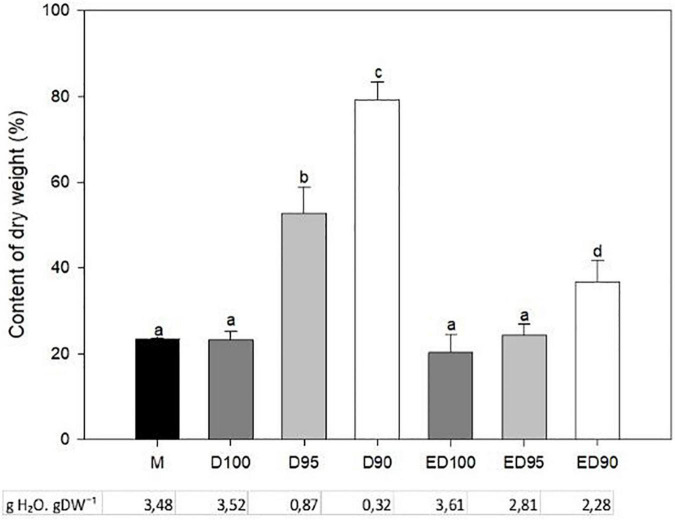
The effect of desiccation on the dry weight and water content of mature embryos (M), control embryos at half (D100), and at the end (ED100) of treatment with 100% relative humidity, and embryos exposed to relative humidity of 95 and 90% during the first half of desiccation (D95 and D90) and subsequently transferred to 100% relative humidity (ED95 and ED90). Values are given as means ± standard deviations of four independent experiments (*n* = 8). Different letters indicate a statistically significant difference at *p* ≤ 0.05.

In control embryos exposed to 100% relative humidity, DW remained unchanged (20–23%) throughout the desiccation process (Figure 4, ED100). Reducing the relative humidity to 95 or 90% in the first half of desiccation caused intense water loss in the embryos, which was reflected in a sharp increase in the dry matter content. DW doubled in D95 and even tripled in D90 embryos. When D95 and D90 embryos were returned to high humidity for the rest of the desiccation, DW content in ED95 approached the value of the control embryos (ED100) and remained two-fold higher in ED90 compared to the control variant (Figure 4).

### Malondialdehyde and abscisic acid content

**FIGURE 5:**
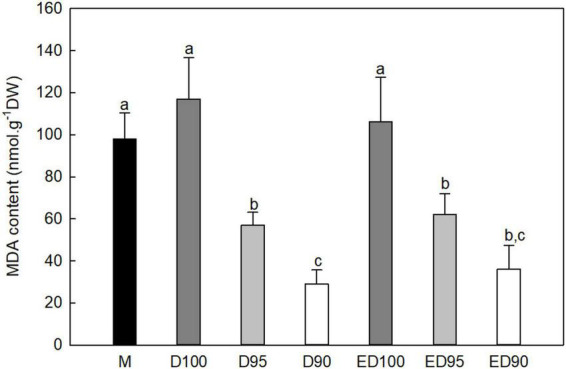
Malondialdehyde (MDA) content in mature embryos (M), control embryos at half (D100) and at the end (ED100) of treatment with 100% relative humidity, and embryos exposed to 95 and 90% relative humidity during the first half of desiccation (D95 and D90) and subsequently transferred to 100% relative humidity (ED95 and ED90). Values are given as means ± standard deviations of four independent experiments (*n* = 8). Different letters indicate a statistically significant difference at *p* ≤ 0.05.

In embryos exposed to high relative humidity (D100), the level of ROS production (measured by the amount of MDA present) increased slightly compared to mature embryos (M; Figure 5). However, a water deficit in embryos desiccated at 95% relative humidity (D95) for 11 days caused a decrease in MDA content by more than 50% and in embryos desiccated at 90% relative humidity (D90) it decreased to 25% of the control values. Although the subsequent transfer to a high relative humidity environment slightly increased the MDA content of embryos, the final values did not exceed 60% (ED95) and 34% (ED90) of the control values (ED100).

The content of ABA and its metabolites was also measured in desiccated embryos and preliminary results showed the expected decrease in ABA levels (Supplementary Figure 1). Treatment at 100 and 95% relative humidity resulted in a sharp decrease in the content of ABA and its metabolites, with ABA glucose ester (ABA-GE) being the most abundant form. Reducing the relative humidity to 90% at the beginning of desiccation substantially affected the metabolism of ABA. The content of ABA and its metabolites was more than five times higher (D90) than in the control embryos (D100), and the major component of ABA metabolites was dihydrophaseic acid (DPA). This pattern was maintained until the end of desiccation (ED90).

### Content of polyamines

**FIGURE 6:**
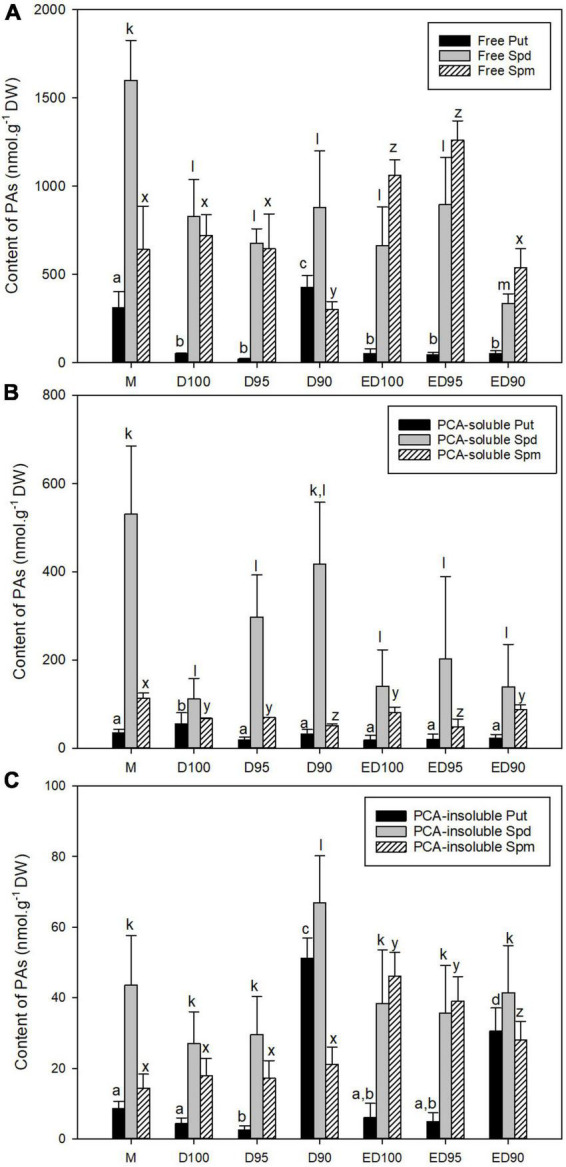
The content of free PAs **(A)**, PCA-soluble PAs **(B)**, and PCA-insoluble PAs **(C)** in mature embryos (M), control embryos at half (D100) and at the end (ED100) of treatment with 100% relative humidity, and embryos exposed to 95 and 90% relative humidity during the first half of desiccation (D95 and D90) and subsequently transferred to 100% relative humidity (ED95 and ED90). Values are given as means ± standard deviations. Bars represent standard deviations of four independent experiments (*n* = 8). Different letters indicate a statistically significant difference at *p* ≤ 0.05. a,b,c,d - Put forms; k,l,m - Spd forms; x,y,z - Spm forms.

In the course of desiccation of embryos, a significant decrease in the total content of PAs was observed. Mature somatic embryos (M) were characterized by higher Spd than Spm content, while Put content represented the lowest proportion of the sum of free, soluble, and insoluble conjugates of PAs (Supplementary Figure 2). In embryos subjected to control conditions with relative humidity of 100% (D100 and ED100), the content of free Put and Spd gradually decreased, whereas the content of Spm increased and was significantly higher than Spd at the end of the experiment in ED100 (Figure 6A). Lower relative humidity of 95% had no significant effect on the PA content of embryos (D95). However, drier air at 90% relative humidity caused a 50% decrease in Spm content in embryos (D90) and a more than eightfold increase in Put content (Figure 6A). After these embryos were subsequently exposed to 100% relative humidity (ED90), free Put content decreased to levels comparable to ED100 and ED95, but both free Spd and Spm content decreased significantly to 50% (compared to ED100).

The contents of PA conjugates were lower than those of free PAs. The significantly higher contents of Spd conjugates determined in D95 and D90 were the only obvious differences from those determined in the control (Figures 6B,C). The bound PAs accounted for the smallest fraction of the total pool of PAs. When embryos were exposed to 90% relative humidity, the content of bound forms of Put and Spd increased eleven-fold and 2.5-fold, respectively, compared to control conditions (D100). The fivefold increase in Put content persisted in ED90 compared to ED100.

As desiccation progressed, changes in the individual PAs resulted in changes in the ratios of Spd/Put, Spm/Put, and Spd/Spm (Supplementary Figure 3). The decrease in free Put content in D100 and D95 embryos resulted in an increase in the ratio of Spd/Put and Spm/Put compared with the values in mature embryos. In contrast, in D90 embryos, a significant increase in free and bound Put resulted in a substantial decrease in both the ratio of Spd/Put and Spm/Put. The decrease in free Put and its conjugates in ED90 embryos at the end of desiccation (compared with D90) was reflected in the increase in the ratios of Spd/Put and Spm/Put. The values of Spd/Spm ratios determined in D100, ED100, D95, and ED95 embryos showed a similar character and did not differ from each other. The different Spd/Spm ratios in D90 were caused by relatively high levels of Spd in free, conjugated, and bound forms.

### Activities of ornithine decarboxylase, arginine decarboxylase, and S-adenosylmethionine decarboxylase enzymes during desiccation of somatic embryos

**FIGURE 7:**
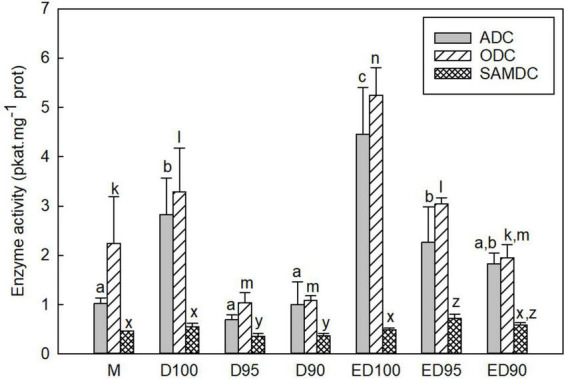
Activities of PA biosynthetic enzymes ADC, ODC, and SAMDC in mature embryos (M), control embryos at half (D100) and at the end (ED100) of treatment with 100% relative humidity, and embryos exposed to 95 and 90% relative humidity during the first half of desiccation (D95 and D90) and subsequently transferred to 100% relative humidity (ED95 and ED90). ADC, arginine decarboxylase; ODC, ornithine decarboxylase; SAMDC, S-adenosylmethionine decarboxylase. Values are given as means ± standard deviations. Bars represent standard deviations of three independent experiments (*n* = 6). Statistical analyzes were performed for the activities of each enzyme. Different letters indicate a statistically significant difference at *p* ≤ 0.05. a,b,c - ADC; k,l,m,n - ODC; x,y,z - SAMDC.

The activities of the three enzymes responsible for the biosynthesis of the major PAs were measured in the soluble and particulate fractions; the results are presented by the sum of the activities of both fractions (Figure 7). In embryos during desiccation, both ADC and ODC activities were responsible for Put biosynthesis. In embryos exposed to control conditions, the variations in the activities of ADC and ODC were very similar during the desiccation period; both activities increased slightly during the 3-week period in control embryos (D100 and ED100; Figure 7). Embryos desiccated at reduced relative humidity showed a decrease in enzyme activities (D95 and D90). Exposure to reduced relative humidity resulted in a decrease in ADC activities to 24 and 35% and ODC activities to 31 and 33% in D95 and D90, respectively, compared with control D100. Subsequent transfer to 100% relative humidity resulted in a significant increase in both activities: ADC to 51 and 41% and ODC to 58 and 37% in ED95 and ED90, respectively. Only moderate changes in SAMDC activity were observed in embryos exposed to control conditions, whereas embryos desiccated at reduced relative humidity showed a slight decrease in enzyme activity (D95 and D90). Subsequent transfer of embryos to 100% relative humidity increased SAMDC activity. At the end of desiccation, the ED95 and ED90 embryos reached 146 and 120%, respectively, of the SAMDC activity determined in the ED100 control.

### Cell wall modifying enzymes

**FIGURE 8:**
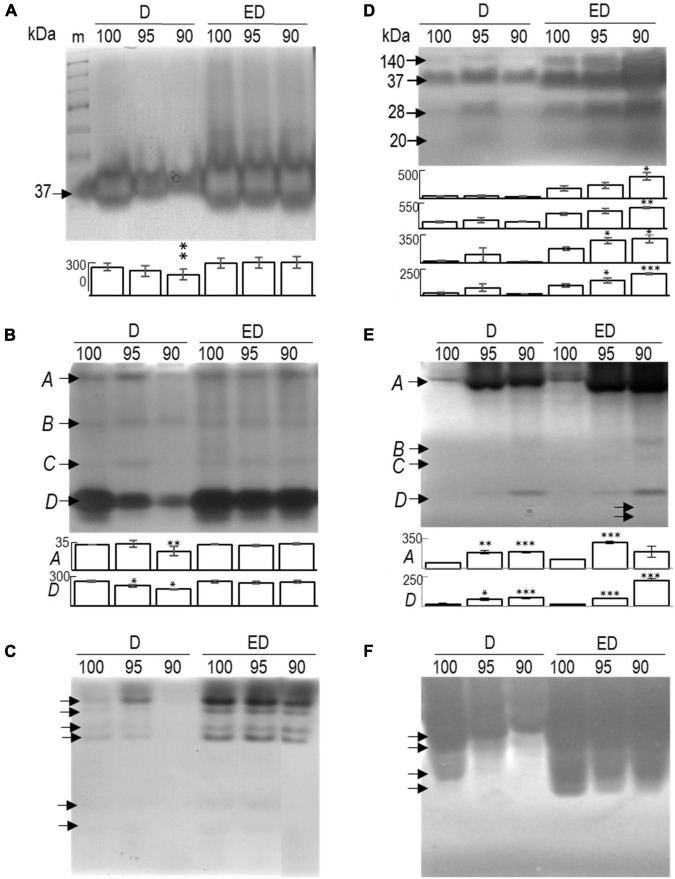
Separation and in-gel detection of β-1,3-glucanases **(A–C)** and chitinases **(D–F)** in mature embryos (M), control embryos at half (D100), and at the end (ED100) of treatment with 100% relative humidity, and embryos exposed to 95 and 90% relative humidity during the first half of desiccation (D95 and D90) and subsequently transferred to 100% humidity (ED95 and ED90). The overall profiles of total β-1,3-glucanases **(A)** and chitinases **(D)** after separation in SDS-PAGE with specific enzyme substrates and separation of acidic/neutral **(B,E)** and basic/neutral **(C,F)** isoforms of β-1,3-glucanases and chitinases. The activity values of the isoforms that were significantly affected by the applied conditions (compared with the corresponding control variants) are indicated in the graphs below the gel images (from top to bottom). *P < 0.05; **P < 0.01; and ***P < 0.001. Data represent the means ± standard deviations. m, molecular marker.

Gel separation based on protein size (SDS-containing gels) and subsequent enzyme detection identified a single fraction of ∼37 kDa β-1,3-glucanases in mature (not shown) somatic embryos. Under control conditions, their activity remained unaffected, whereas desiccation at reduced relative humidity markedly suppressed their activity. However, when embryos were subsequently transferred to a 100% relative humidity environment, strong activation of the enzyme was observed in embryos exposed to 95 and/or 90% relative humidity during the first half of desiccation (Figure 8A). This pattern apparently involves the acidic isoforms; further separation under native conditions confirmed a similar response of two of the four acidic isoforms detected (Figure 8B, isoforms *A* and *D*). The activity of all four basic enzyme fractions was pronounced during the second half of desiccation (Figure 8C).

The enzyme profile of chitinases in all embryos (including matured embryos, data not shown) comprised four different isoforms of ∼140, 37, 28, and 20 kDa (Figure 8D). Their activities during the first 11 days of desiccation appeared to be inconsistent for unknown reasons (Figures 8D,E). However, in response to transfer to 100% relative humidity, each of them was statistically significantly induced in at least one variant – typically in embryos exposed to 90% relative humidity during the first half of desiccation (ED90; Figure 8D). With separation under native conditions, we detected several acidic/neutral (Figure 8E), and basic/neutral isoforms (Figure 8F). The activity of the largest acidic fraction gradually increased at reduced humidity during the first 11 days (D95 and D90) and later when desiccation continued at 100% relative humidity (ED95 and ED90; Figure 8E, isoforms *A* and *D*). Most of the chitinase activity in the embryos likely came from the basic isoforms (Figure 8F); unfortunately, because of their very high activity, we could not quantify their activities.

### Expression of genes for polyamine biosynthetic enzymes

**FIGURE 9:**
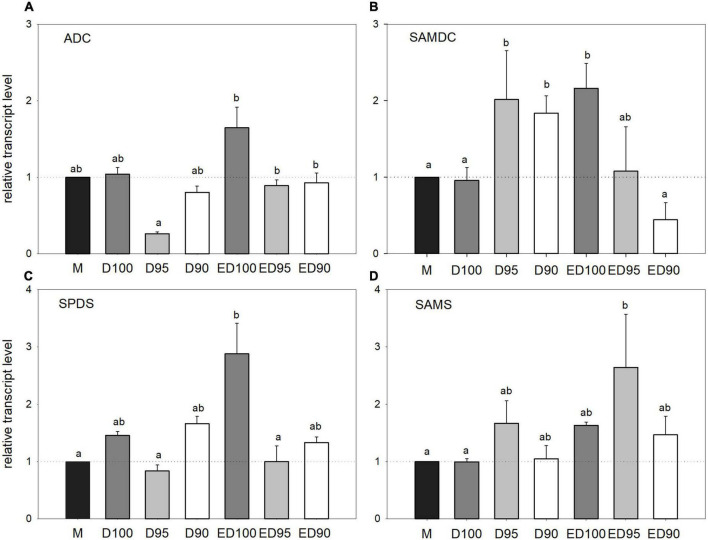
Relative transcript levels of polyamine biosynthetic enzyme genes ADC **(A)**, SAMDC **(B)**, SPDS **(C)**, and SAMS **(D)** in mature embryos (M), control embryos at half (D100), and at the end (ED100) of treatment with 100% relative humidity, and embryos exposed to 95 and 90% relative humidity during the first half of desiccation (D95 and D90) and subsequently transferred to 100% relative humidity (ED95 and ED90). Transcript levels are compared relative to expression levels in mature embryos (value1). Values are expressed as means ± standard deviations (*n* = 9). Different letters indicate statistically significant difference at *p* ≤ 0.05. ADC, arginine decarboxylase; SAMDC, S-adenosylmethionine decarboxylase; SPDS, spermidine synthase; and SAMS, S-adenosylmethionine synthase.

The expression levels of four polyamine biosynthetic enzyme genes were followed by qPCR analysis during the desiccation of embryos at different humidity levels. The expression level of the ADC gene (Figure 9A) increased slightly under control conditions throughout the desiccation period (D100 and ED100). Desiccation at reduced humidity caused a slight decrease in transcription that was significant only in D95 embryos. In the course of treatment at high relative humidity conditions, the expression level of SAMDC doubled (Figure 9B, ED100). At reduced relative humidity (D95 and D90), this increase in SAMDC transcript levels became less pronounced and returned to the levels found in mature embryos at the end of desiccation (ED95 and ED90). The relative level of SPDS gene transcript increased steadily during treatment under control conditions (Figure 9C, D100 and ED100), whereas it remained unchanged at lower relative humidity levels (D95 and D90) throughout the experiment (ED95 and ED 90). The expression of S-adenosylmethionine synthase gene was not affected by any desiccation treatment (Figure 9D); a slight but not significant induction was observed in ED95 embryos.

### Expression of genes for cell wall modifying enzymes

**FIGURE 10:**
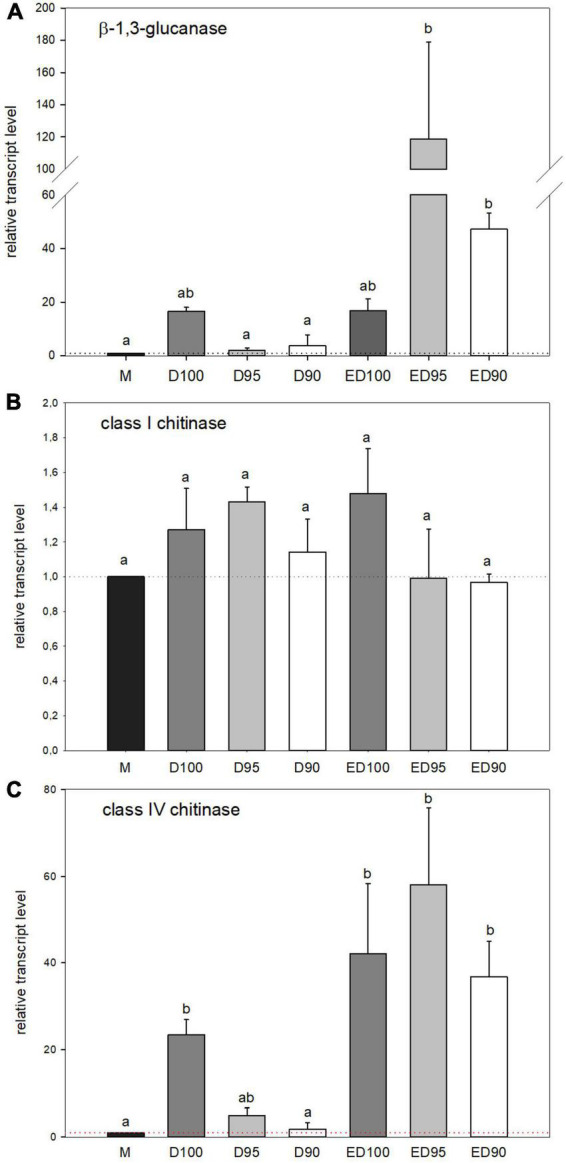
Relative transcript level of β-1,3-glucanase **(A)**, class I chitinase **(B)**, and class IV chitinase **(C)** in mature embryos (M), control embryos at half (D100) and at the end (ED100) of treatment with 100% relative humidity, and embryos exposed to 95 and 90% relative humidity during the first half of desiccation (D95 and D90) and subsequently transferred to 100% relative humidity (ED95 and ED90). Transcript levels are compared relative to expression levels in mature embryos (value1). Values are expressed as means ± standard deviations (*n* = 9). Different letters indicate a statistically significant difference at *p* ≤ 0.05.

The expression of the β-1,3-glucanase gene studied was affected as a function of humidity (Figure 10A). Treatment with 100% relative humidity resulted in a 16-fold increase in expression (D100) compared to mature embryos (M), remained until the end of the experiment (ED100). In embryos desiccated at lower relative humidity during the first 11 days (D95 and D90), gene expression remained unchanged compared to mature embryos (M), but increased sharply as desiccation progressed, reaching 7-fold (ED95), and 3-fold (ED90) levels compared to control ED100.

As for chitinases, the expression of a single class I chitinase gene and a single class IV chitinase gene were analyzed. The expression of the class I chitinase gene was not affected by any desiccation treatment (Figure 10B), in contrast to the class IV chitinase gene (Figure 10C). During treatment with 100% relative humidity, the expression of the class IV chitinase gene increased continuously compared to the transcript level in mature embryos (M). In the first half of the treatment (experiment; D100) a 20-fold higher transcript level was detected, which was further pronounced up to 40-fold higher transcript abundance at the end of the experiment (ED100). Reduced relative humidity in the first half of desiccation negatively affected expression of the class IV chitinase gene (D95 and D90), whereas transfer to 100% relative humidity restored its expression (ED95 and ED90) to the level observed in control embryos (ED100).

## Discussion

The conversion of somatic embryos into high-quality emblings depends on the development of functional root and shoot systems (Hazubska-Przybyl et al., 2015). To improve the efficiency of germination, special procedures are used, such as partial drying of somatic embryos at high relative humidity (Roberts et al., 1990; Find, 1997) or low temperature treatment (Tikkinen et al., 2018b). For some species and cell lines, including *P. abies* (Tikkinen et al., 2018a), germination can occur without desiccation, but in general, desiccation is thought to improve germination rates and subsequent plant formation (Egertsdotter et al., 2019). Desiccation tolerance of conifer somatic embryos differs among species and genotypes and depends on the previous conditions of maturation (Hay and Charest, 1999). In all tested embryogenic cultures recently induced in our laboratory, desiccation of embryos at high relative humidity (100%, Figure 2) increased the germination rate compared with embryos germinated immediately after maturation. The desiccation treatment had a dual effect on somatic embryos’ further development depending on the humidity level – it positively affected embling germination and growth (Figures 2, 3), while causing stress due to water loss in embryos (Figure 4).

The intensity of partial drying and thus the impact of different relative humidity during the desiccation step determines the extent and outcome of these two possible effects of desiccation. ABA probably plays an important role in these processes, as it is a key regulator of plant responses to stresses and of plant growth and development. Degradation of ABA has been associated with the release of seed dormancy (Finch-Savage and Leubner-Metzger, 2006; Vondrakova et al., 2020), and decreased ABA content has been detected in spruce somatic embryos prior to germination (Find, 1997). The expressive change in ABA content in embryos during desiccation compared to mature embryos is likely due to the maturation medium being supplemented with a relatively high concentration of ABA that is omitted during desiccation (Vondrakova et al., 2018). Then, the differences in ABA content between embryos desiccated at different relative humidity correlate with the ability of the embryos to metabolize ABA (remaining in the embryos after maturation). At the same time, synthesis of ABA can be induced by stress conditions. Our preliminary data on ABA (Supplementary Figure 1) showed higher levels of ABA and ABA metabolites in embryos exposed to 90% relative humidity. The higher levels of ABA and DPA, the final product of the ABA hydroxylation pathway, suggest that embryos are unable to catabolize successfully the huge amount of ABA present after maturation. In contrast, in embryos from all other treatments, the prevailing ABA metabolite was ABA-GE, which is thought to be a reversibly inactive form of ABA (Seo and Marion-Poll, 2019). The reduced degradation rate of ABA may be related to the low water content in embryos exposed to 90% relative humidity and the consequent slowing of the enzymatic ABA degradation machinery.

As somatic embryos are exposed to osmotic stress during desiccation, ROS could accumulate excessively, leading to lipid peroxidation and damage to cellular component (Mailloux and Harper, 2011). During the experiments, the changes in MDA levels were tested as a marker of the peroxidation of membrane lipids to assess the extent of ROS production. The changes in MDA levels in embryos desiccated at 100% relative humidity were small, indicating adequate antioxidant protection (Figure 5). We hypothesized that the reduced humidity applied during desiccation would increase MDA values; however, the opposite was true, the applied reduced humidity negatively affected MDA content in somatic embryos. Activation of different antioxidant defense systems in response to the lower humidity could lead to efficient scavenging of ROS predominating over their production, which could explain the lower MDA levels in D95 and D90 embryos.

Polyamines are essential biomolecules with a dual role in plants – they are known to be involved in the regulation of many plant developmental and growth processes as well as in the response to various environmental stimuli (Wuddineh et al., 2018). Our findings indicate the stress-induced changes in PA homeostasis observed in desiccated embryos. Reduced humidity during desiccation resulted in changes in the free, conjugated, and bound forms of PAs compared to control embryos (Figure 6). A markedly increased content of free Put in D90 in spite of decreased ADC and ODC activities (Figure 7) and a sharp decrease in the content of free Spm indicate that free Spm could be converted back to Spd and Spd to Put. Reconversion is the opposite reaction to PA biosynthesis and is catalyzed by the enzyme polyamine oxidase (Kusano et al., 2008). Changes in PA levels in response to various abiotic stress conditions are often accompanied by stimulation of PA oxidation (Kamada-Nobusada et al., 2008; Tiburcio et al., 2014), but knowledge of the physiological function(s) of PA catabolic enzymes and their involvement in PA homeostasis is still insufficient (Takahashi et al., 2018).

A considerable proportion of PAs in plant cells can be diverted from physiological activity by conjugation with soluble phenolic acids or with macromolecules (Grienenberger et al., 2009; Fellenberg et al., 2012). The significantly higher content of Spd conjugates in both D95 and D90 (Figures 6B,C) suggests that PA conjugation in our system is involved in PA homeostasis in addition to oxidative deamination. In general, the accumulation of bound and conjugated PAs is an important protective property in plants under adverse environmental conditions (Yu et al., 2019). It has been reported that cell wall-bound PAs mediate the formation of bonds between individual cell wall components and between the cell wall and the cell membrane (Berta et al., 1997). Changes in the content of conjugated and bound PAs have been found in the adaptation of vetiver grass to water deficit (Zhou and Yu, 2010), and the drought resistance of rice cultivars has been associated with obviously induced increases in bound PAs (Yang et al., 2007). The response to water deficit in triticale cultivars was also associated with a gradual increase in cell wall-bound PAs (Hura et al., 2015). The observed increase in conjugated and bound Spd content, and especially bound Put content in D90, is consistent with these results. Although mediation of bond formation is attributed to Spm, similar involvement of Put and thermospermine in the defense response to, e.g., biotic stress has been proposed (Jimenez-Bremont et al., 2014).

Free PAs have been studied more extensively than the soluble conjugated and/or bound forms (Chen et al., 2019). An inadequate Spd/Put ratio has also been considered an important factor in SE and has been associated with abnormal growth and disordered cell proliferation in grape somatic embryos (Faure et al., 1991). Higher values of the ratio of free forms Spd/Put and Spm/Put determined in D100 and D95 (relative to M, Supplementary Figure 3) were caused by low levels of free Put and are characteristic of Norway spruce somatic embryos during the desiccation period (Gemperlova et al., 2009). However, in the case of D90, the situation was different because both ratios of free forms, Spd/Put and Spm/Put, were very low due to the significantly increased content of free Put. Unfortunately, the physiological significance of these changes in PA ratios is still unclear (Huang et al., 2004; Murray-Stewart et al., 2018).

Like PAs, the cell wall-modifying enzymes chitinases and β-1,3-glucanases play multiple roles in plant development and responses to various stresses (Perrot et al., 2022; Vaghela et al., 2022). In conifer SE, their role has been demonstrated for example in white spruce (Dong and Dunstan, 1997; Gao et al., 2022), black pine and hybrid firs (Fráterová et al., 2013), and Norway spruce (Dyachok et al., 2002). Some of the β-1,3-glucanases are active during seed maturation and have been shown to initiate rupture of seed testa during germination (Leubner-Metzger, 2005). Remarkably, both enzymes are considered components of the second line of defense against abiotic stresses, including water deprivation. The presence of ∼37 kDa β-1,3-glucanase in Norway spruce somatic embryos (Figure 8A) is consistent with previously detected glucanases of similar size in chicory (Helleboid et al., 1998, 2000), pea (Buchner et al., 2002), or Scots pine (Pirttilä et al., 2002). Water loss during desiccation inhibited the activities of two (acidic) β-1,3-glucanases in the embryos (Figure 8B; isoforms *A, D*) as well as the expression of a single β-1,3-glucanase gene (Figure 10). This probably led to the deposition of the enzyme substrate – callose. The deposition of callose is the result of a shift in the balance between the degrading (β-1,3-glucanase) and the synthesizing enzyme (β-1,3-glucan synthase, also called callose synthase; van der Schoot et al., 2014). Callose forms plasmodesmal “clamps” that modulate the movement of molecules across plasmodesmata in response to developmental and environmental stimuli (De Storme and Geelen, 2014), including water deprivation (Gregorová et al., 2015). In many dormant systems, callose clogs sieve tubes as well as plasmodesmata in the shoot apical meristem, isolating the meristem from surrounding tissues (van der Schoot et al., 2014). In agreement with this, β-1,3-glucanase activities in desiccated Norway spruce embryos depended on relative humidity. Enzyme activity data were consistent with the massive expression of the studied β-1,3-glucanase gene in the second half of desiccation, probably enabling rapid symplastic transport.

We detected chitinases of four different sizes (20, 28, 37, and 140 kD) in Norway spruce somatic embryos (Figure 8). Previously, 25, 48, and 56 kDa chitinases were detected in *P. caribaea* (Domon et al., 2000) and more than six different isoforms were present in callus culture of *Pinus sylvestris* (23, 25, 31, 36.5, 75, and some ≥ 124 kDa; Pirttilä et al., 2002). In our experiment, reduced relative humidity induced at least a single acidic chitinase in embryos, whereas subsequent transfer to 100% relative humidity activated several basic and some acidic isoforms. Their putative role is the release of signaling lipochitooligosaccharides from arabinogalactan proteins in different embryo structures (Dyachok et al., 2002; Zieliński et al., 2021), which are thought to play a role in morphogenesis and defense (Rojas-Herrera and Loyola-Vargas, 2002). The molecular function of chitinases in embryos could also include antifreeze activity (Żur et al., 2013), cell wall strengthening/loosening under changing water conditions (Gregorová et al., 2015), and cell wall lysis of pathogens (Grover, 2012) or endophytes (Pirttilä et al., 2002). Expression of the class IV chitinase gene was strongly induced in Norway spruce embryos desiccated at high relative humidity (Figure 10), in contrast to the previously described class IV chitinase (Hietala et al., 2004). This suggests that this gene likely plays a role in the late stages of embryo development, or in seed adaptation to changing humidity or both. In contrast, the activity of the class I chitinase gene was not affected in our experiment. Previously, a class I chitinase was induced by low temperature in mature white spruce somatic embryos (Gao et al., 2022), but was suppressed by wounding (Hietala et al., 2004). Chitinases of different classes were also differentially expressed in *P. glauca* during the transition between seasonal activity and dormancy (González et al., 2015).

While chitinases likely alter cell wall elasticity (Yokoyama and Nishitani, 2004) and prevent the invasion of microbes naturally present in the soil, glycoside-hydrolyzing glucanases could affect water trafficking and/or the accumulation of soluble sugars for osmotic adjustment (Levy et al., 2007). These functions are consistent with the accumulation of PAs studied and suggest the activation of mechanisms to adapt the embryos to changing moisture conditions, possibly to avoid cell death (Wiweger et al., 2003) and ensure better survival.

## Conclusion

Desiccation at high relative humidity promoted germination of Norway spruce somatic embryos, increased the activities of ADC and ODC, the activities of β-1,3-glucanases and transcription of β-1,3-glucanase and class IV chitinase genes. Exposure of matured somatic embryos to decreased humidity levels resulted in stress-induced changes in PA homeostasis as well as the content of ABA and its metabolites. The adaptation of embryos desiccated at reduced relative humidity resulted in changes in the content of free, conjugated, and bound forms of PAs. A high rate of embryo dehydration elicited a significant increase in the content of free Put, although the activities of ADC and ODC decreased, which together with a sharp decrease in the content of free Spm indicated reconversion of free Spm. The increase in bound PAs was triggered by adaptation to a severe water deficit (90% relative humidity), confirming the role of PAs in strengthening the cell wall under environmental stress conditions. Low-humidity desiccation also markedly suppressed the activities of cell wall-modifying enzymes β-1,3-glucanases and chitinases, as well as the expression of β-1,3-glucanase and class IV chitinase genes. Most of the monitored enzyme activities were temporarily attenuated by low relative humidity, but were restored after the water deficit conditions passed away. Desiccation at lower relative humidity appears to impede the processes leading to physiological maturity and subsequent ability of the embryos to develop into emblings.

## Data availability statement

The original contributions presented in the study are included in the article/Supplementary materials, further inquiries can be directed to the corresponding author.

## Author contributions

MC, IM, and ZV had the main responsibility of planning and experimental set up for the study. MC and LG were responsible for MDA analysis, polyamine content and biosynthetic enzyme activity experiments. JMo and ZG did in gel analysis of β-1,3-glucanase and chitinase activities. JMa and VM conducted LC-MS analysis of polyamines and ABA. JK and JP carried out RT-PCR analysis. LF and ZV were responsible for experimental material, obtained micro-morphological data, had main responsibility for data analysis, and for writing of the manuscript draft. KE, IM, ZV, LF, MC, and VM writing—review and editing. MC, IM, and LG funding acquisition. All authors contributed to the article and approved the submitted version.

## Footnotes

1
http://www.scioncorp.com

## Abbreviations

SE: somatic embryogenesis
PAs: polyamines
ROS: reactive oxygen species
Put: putrescine
Spd: spermidine
Spm: spermine
ODC: ornithine decarboxylase
ADC: arginine decarboxylase
SAMDC: S-adenosylmethionine decarboxylase
SPDS: spermidine synthase
SPMS: spermine synthase
MDA: malondialdehyde
ABA: abscisic acid
ABA-GE: abscisic acid glucose ester
DPA: dihydrophaseic acid.
