# Supplementary material for: The humidity level matters during the desiccation of Norway spruce somatic embryos
Source: Front Plant Sci. 2022 Jul 29;13:968982. doi: 10.3389/fpls.2022.968982 (PMC9372446; doi:10.3389/fpls.2022.968982)

**Supplementary Figure 1**. Content of ABA (separately also in detail) and ABA metabolites in mature embryos (M control embryos at half (D100) and at the end (ED100) of desiccation at 100% relative humidity, and embryos exposed to 95% and 90% relative humidity during the first half of desiccation (D95 and D90) and subsequently transferred to 100% relative humidity (ED95 and ED90). ABA – abscisic acid; ABA-Me – abscisic acic methyl ester; ABA-GE – ABA-glucose ester; PA – phaseic acid; DPA – dihydrophaseic acid; 7OH-ABA – 7–hydroxy-ABA; 9OH-ABA – 9-hydroxy-ABA; NeoPA – neophaseic acid.


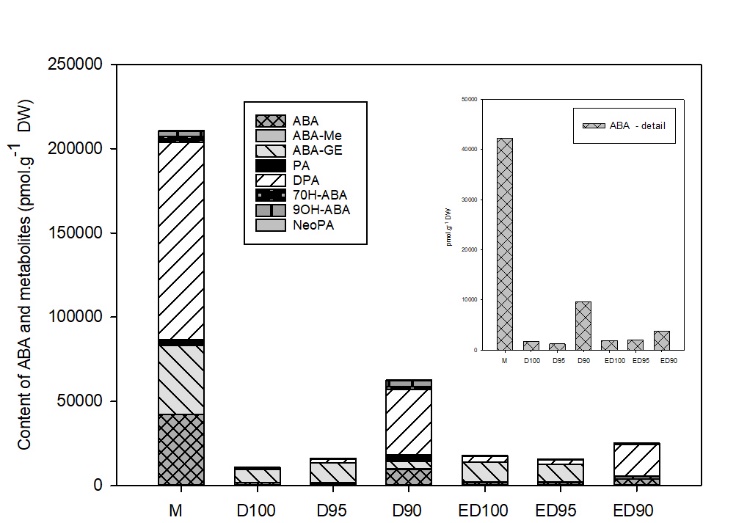

Supplement: Supplementary file 1 [file Data_Sheet_1.docx]
