# Supplementary material for: The humidity level matters during the desiccation of Norway spruce somatic embryos
Source: Front Plant Sci. 2022 Jul 29;13:968982. doi: 10.3389/fpls.2022.968982 (PMC9372446; doi:10.3389/fpls.2022.968982)

**Supplementary Figure 2.** The total content of PAs in mature embryos (M), in control embryos at half (D100) and at the end (ED100) of desiccation at 100% relative humidity, and embryos exposed to 95% and 90% relative humidity during the first half of desiccation (D95 and D90) and subsequently transferred to 100% relative humidity (ED95 and ED90). Values represent the sum of free, PCA soluble, and PCA insoluble PAs; Put in red, Spd in blue, and Spm in green colors. (These data are included separately in Fig. 6, where they are expressed as means ± standard deviations with bars representing SD of four independent experiments (n=8) and different letters indicating statistically significant difference at p≤0.05.)


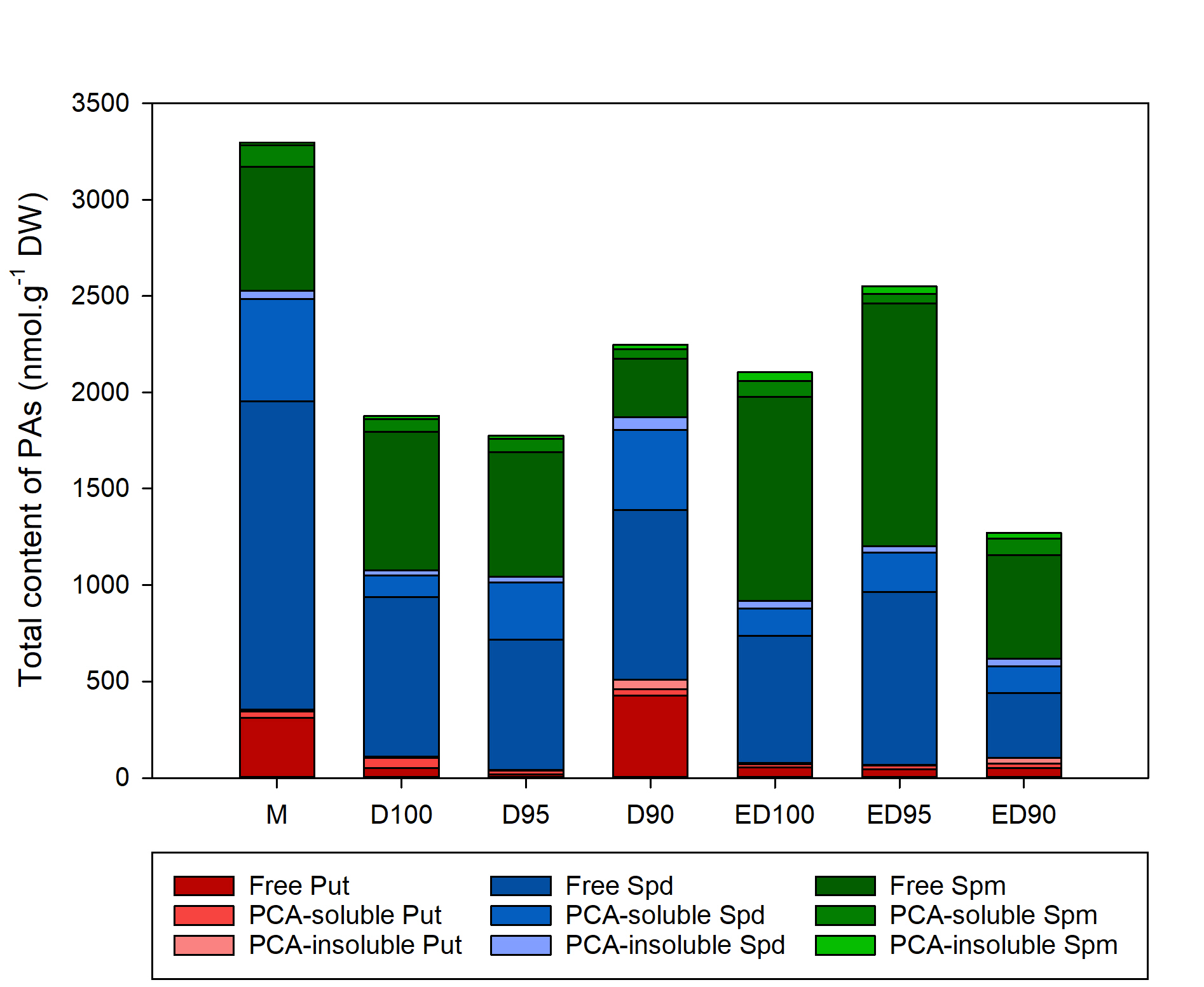

Supplement: Supplementary file 2 [file Data_Sheet_2.docx]
