# Supplementary material for: The humidity level matters during the desiccation of Norway spruce somatic embryos
Source: Front Plant Sci. 2022 Jul 29;13:968982. doi: 10.3389/fpls.2022.968982 (PMC9372446; doi:10.3389/fpls.2022.968982)

**Supplementary Figure 3.** The changes in Spd/Put, Spm/Put and Spd/Spm ratios during embryo desiccation in mature embryos (M), control embryos at half (D100) and at the end (ED100) of desiccation at 100% relative humidity, and embryos exposed to 95% and 90% relative humidity during the first half of desiccation (D95 and D90) and subsequently transferred to 100% relative humidity (ED95 and ED90). Values are expressed as means ± standard deviations. Bars represent SD of four independent experiments (n= 8). Different letters indicate statistically significant difference at p≤0.05.


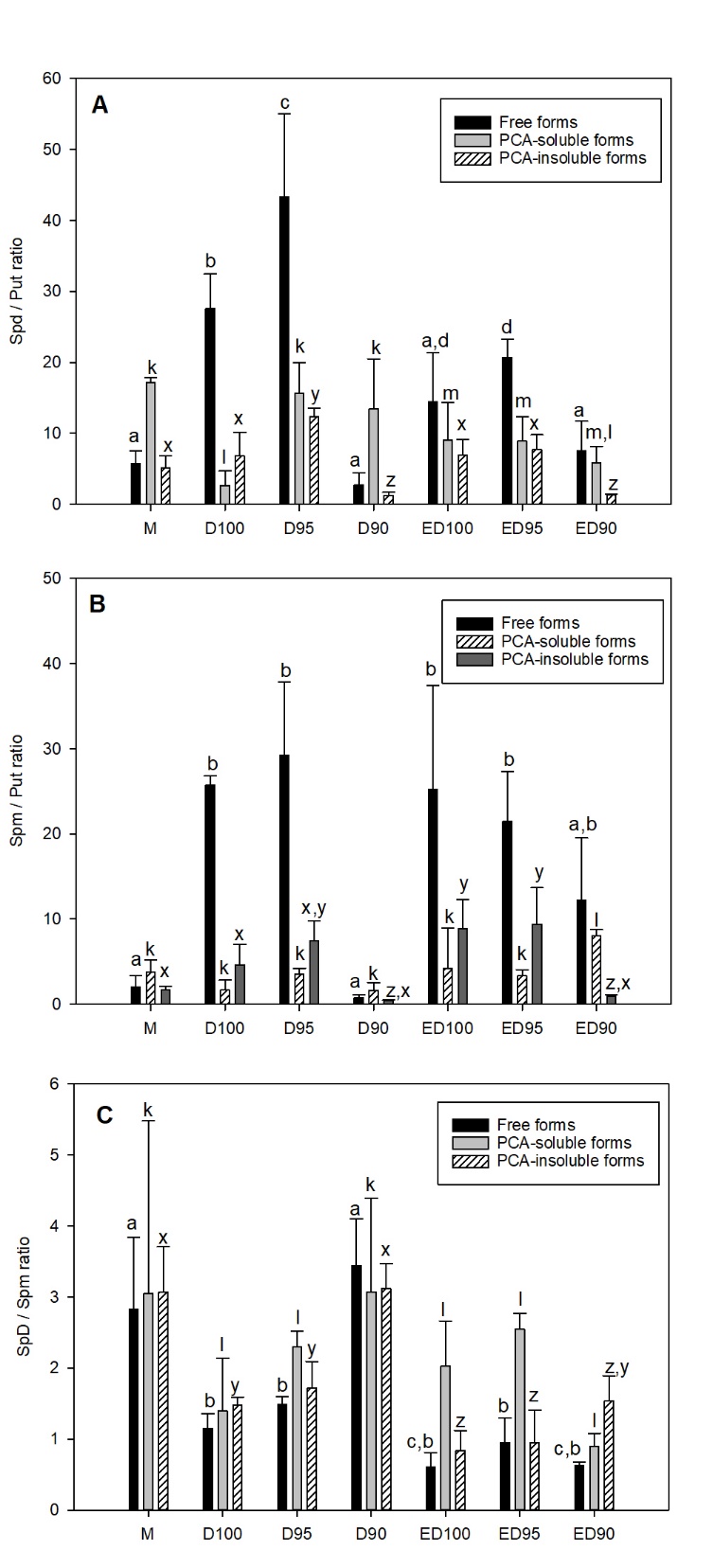

Supplement: Supplementary file 3 [file Data_Sheet_3.docx]
